# Sustainable nanocellulose-supported ZIF-8/ZnO/activated carbon heterostructures enhance charge separation for efficient photocatalytic dye remediation

**DOI:** 10.1038/s41598-026-47425-6

**Published:** 2026-05-02

**Authors:** Amal A. Nassar, Ayman K. El-Sawaf, A. O. Ali, M. F. Mubarak.M

**Affiliations:** 1 Department of Chemistry, College of Science and Humanities, Prince Sattam bin Abdulaziz University, Alkharj, 11942 Saudi Arabia; 2 Production Department, Egyptian Petroleum Research Insitute,Ahmed El-Zomer st., Nasr City, 11727, Cairo, Egypt; 3 Petroleum Application department, Egyptian Petroleum Research Institute, Ahmed El-Zomer st, Nasr City, Cairo, 11727 Egypt

**Keywords:** ZIF-8, ZnO, Activated carbon, Photocatalytic degradation, Methylene blue, Methyl orange, Wastewater treatment, Chemistry, Environmental sciences, Materials science, Nanoscience and technology

## Abstract

The presence of synthetic dyes such as Methylene Blue (MB) and Methyl Orange (MO) in water sources presents a significant environmental issue, attributed to their stability and toxic properties. This study presents the fabrication and evaluation of a multifunctional ZIF-8/ZnO/Activated Carbon/Nanocellulose composite for its photocatalytic efficiency under optimized conditions. The characterization results confirmed the development of a hierarchical porous structure characterized by an increased surface area, enhanced light absorption, and improved charge separation. Photocatalytic degradation tests demonstrated that the composite attained maximum efficiencies of 91.8% for MB and 87.4% for MO under optimal conditions of pH 7, a catalyst dose of 0.3 g/L, a temperature of 30 °C, and 60 min of UV irradiation. The adsorption behavior conformed to the Langmuir model, exhibiting high monolayer capacities of qmax (53.0 mg/g for MB and 50.1 mg/g for MO), which indicates a strong affinity between the composite and the dye molecules. Kinetic analyses indicated a pseudo-second-order mechanism, and thermodynamic findings validated the spontaneous and exothermic characteristics of the process. The composite exhibited strong stability across five cycles, demonstrating negligible efficiency loss. The interaction between ZIF-8, ZnO, activated carbon, and nanocellulose markedly improved adsorption, charge transfer, and radical generation, rendering this composite an efficient material for sustainable wastewater treatment .

## Introduction

Water pollutions originating from the discharge of synthetic dyes such as Methyl Orange (MO) and Methylene Blue (MB) have become a widespread environment issues^1,2^.They are widely applied in textile, leather and paper industries but their disposal into the environment is a threat to the animals and aquatic ecosystem^3,4^.These dyes are chemically stable, nondegradable and have long-term persistence in the natural waters; thus create bioaccumulation and ecosystem imbalance. MB exposure leads to various human health problems such as eye irritation, respiratory difficulty, and gastrointestinal toxicity, whereas MO and its degradation intermediates pose mutagenic and carcinogenic risks^1,5^.Moreover, even when dye concentrations are low, they can inhibit light penetration in bodies of water and therefore inhibit photosynthesis and cause disruption to the aquatic food chains. However, conventional treatments for dye removal including adsorption, coagulation and biological processes have drawbacks in treatment efficiency, cost of treatment and environmental impact^6^. Therefore, there is an urgent requirement to develop a new generation of environmentally friendly and efficient technologies for the degradation of dye-polluted water^7^.

Photocatalytic​‍​‌‍​‍‌ degradation is becoming an outstanding approach to remove organic contaminants from water^8,9^.The method uses semiconductor materials that can produce redox reactions upon illumination, which decays the organic contaminants into safe intermediates^10^. Zinc oxide (ZnO) is a material known for its high photochemical activity. Unfortunately, ZnO possesses low specific surface area and is photounstable under UV light illumination, thus limiting its practical applications^11,12^.Accordingly, researchers have been attempting to improve the efficiency of ZnO by combination with other substances such as AC and NC in order to overcome these ‍​‌‍​‍‌ drawbacks^13,14^.

Metal–Organic Frameworks (MOFs) are crystalline porous materials constructed from metal ions or clusters coordinated with organic ligands, forming highly ordered three-dimensional networks. MOFs are characterized by their simply high surface areas, tunable pore structures and adjustable chemical functionalities^15^. Thus, they offer abundant accessible active sites and allow the mass transfer of pollutants, which render MOFs applicable for dye removal compared to many conventional adsorbents and catalysts. When combined with semiconductors, MOFs can improve dye adsorption, light harvesting and charge separation in photocatalytic systems^15^.

Among MOF, Zeolitic Imidazolate Frameworks (ZIF-8) is highly porous and possesses a large specific surface area for the adsorption of organic contaminants^16,17^. Compared with traditional porous materials such as activated carbon alone, ZIF-8 offers a more uniform pore distribution and tunable framework chemistry, enabling stronger interactions with dye molecules^18^. The high porosity possessed by ZIF-8 facilitates dye molecules and adsorbed oxygen to migrate into the photocatalytic active sites, which is conducive for enhancing the activity of dye degradation^16^. Additionally, ZIF-8 is stable under a range of environmental conditions, making it a durable and reliable component for long-term use in wastewater treatment^19^. Its tunable structure also allows for modifications that can optimize its properties for specific applications, such as improving its interaction with various pollutants^16,20^.

Activated carbon (AC) has high adsorption performance due to the relatively large specific surface area and dense micropores^21^. It can effectively capture dye molecules and therefore enhance the photocatalytic degradation process as it could concentrate the pollutants around the active sites of photocatalyst. AC can be regenerated for several times, which is an economical adsorbent for large scale applications^22^. In addition, it can provide more active sites for photocatalytic reactions with ZnO and ZIF-8 together, which can increase the degradation efficiency.

Nanocellulose (NC) is a biodegradable, lightweight and flexible material, having great potential as support for the composite^23^. Its large specific surface area is favorable for the uniform dispersion of ZnO and ZIF-8 particles, ensuring that photocatalytic sites are effectively utilized^24,25^. Unlike purely inorganic supports, NC contributes to mechanical stability while maintaining environmental compatibility, aligning with green chemistry principles^26^. Moreover, NC can be prepared from biomass and is thus an eco-friendly material that meets the new requirement of sustainable technologies in water treatment^27^.

The integration of ZIF-8, ZnO, AC, and NC into a single hybrid composite is designed to achieve a synergistic photocatalytic effect. In this system, ZnO serves as the main photoactive component producing reactive oxygen species (ROS), ZIF-8 amplifies adsorption capacity and contributes in mass transfer, whereas AC augments pollutant concentration and mobility of electron, while NC provides structural stability and prevents nanoparticle aggregation. In summary, the advantages of the incorporation of these functions can overcome the limitations existing in pure ZnO and single phase systems with higher visible light harvesting ability, enhanced charge separation efficiency, larger adsorption capability as well as excellent photocatalytic degradation performance.

The novelty of this study lies in the rational design of a sustainable quaternary ZIF-8/ZnO/AC/NC hybrid composite that integrates adsorption, photocatalysis, charge separation, and structural stabilization within a single multifunctional system. Unlike previously reported binary or ternary ZnO-based composites, this work strategically couples a porous MOF (ZIF-8), a photoactive semiconductor (ZnO), activated carbon for enhanced electron transport, and biodegradable nanocellulose as a green dispersive support. Specifically, the synergistic integration of AC and ZIF-8 creates a unique electron-sink effect that significantly suppresses the rapid charge recombination typically observed in pure ZnO. This hierarchical structure improves dye adsorption, reduces charge recombination, and enhances photocatalytic degradation efficiency for both cationic and anionic dyes, thereby offering a more effective and environmentally compatible approach for wastewater treatment that remains structurally stable due to the NC biopolymer matrix.

The objective of this work is to prepare a new type of hybrid composite composed of ZIF-8 MOF, ZnO, AC, and NC as an efficient photocatalyst for the degradation of organic dyes such as MO and MB. This catalyst is particularly suitable for the present study because it simultaneously integrates adsorption, photocatalysis, structural stabilization, and sustainability within a single multifunctional system, thereby offering a practical and high-performance solution for dye polluted wastewater treatment.

## Experimental

### Materials

Zinc nitrate hexahydrate (≥ 99%, Zn (NO₃)_2_.6H₂O), 2-methylimidazole (≥ 99%, 2-MIM), and 99% methanol were acquired from Sigma-Aldrich and utilized as precursors for ZIF-8. Zinc acetate dehydrate (≥ 98%, Zn (CH₃COO) _2_ 0.2 H₂O), sodium hydroxide (≥ 98%, NaOH), and 99% ethanol were procured from Sigma-Aldrich and utilized as precursors for ZnO. All compounds were utilized as obtained, without additional purification processes. All preparation processes utilized deionized water. Rice straw is sourced from Kafr El Sheikh, Egypt, while coconut shells are obtained from the Red Sea region.

### Synthesis of zeolitic imidazolate frameworks (ZIF-8)

**Fig. 1:**
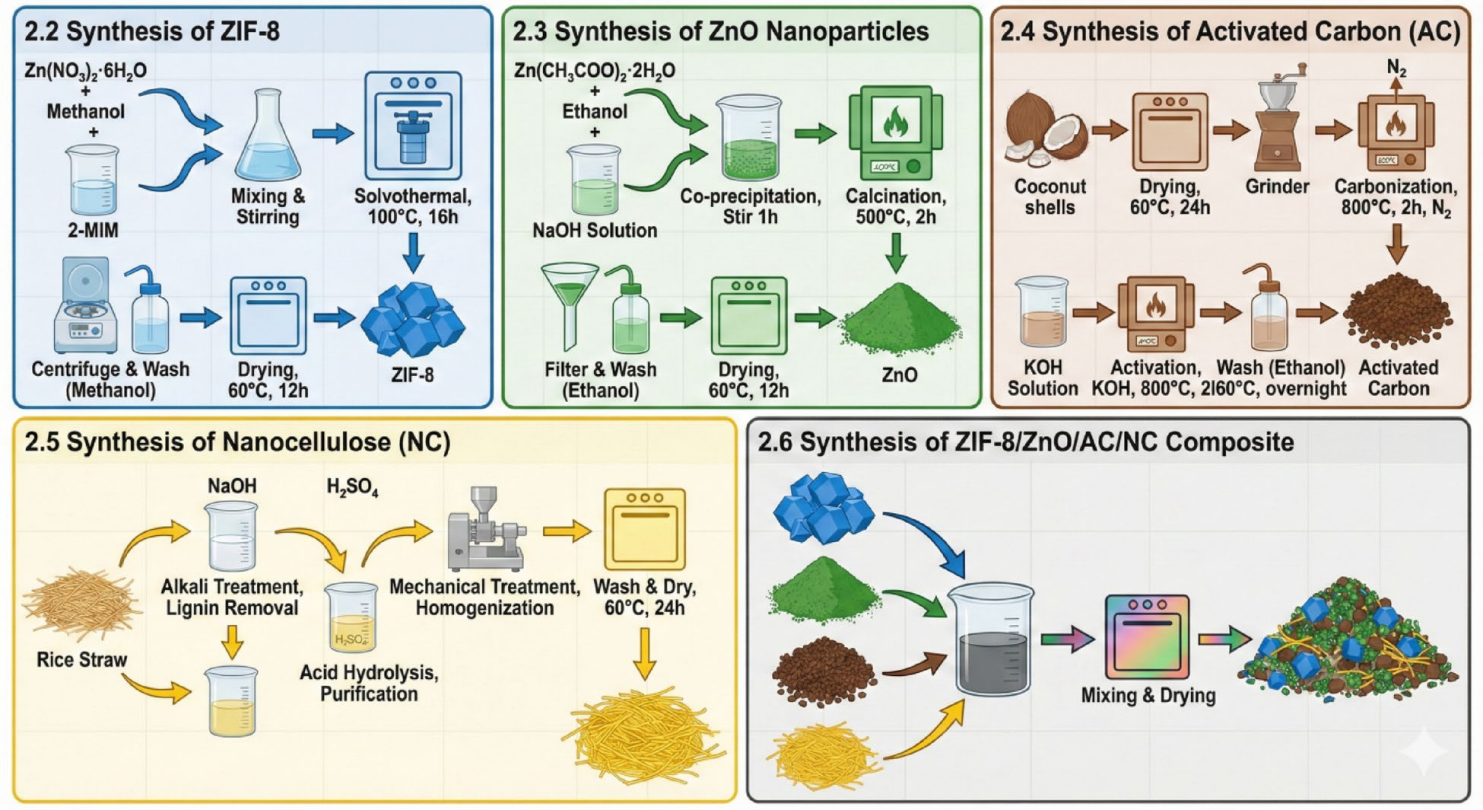
Schematic diagram for the preparation of ZIF-8, ZnO, AC, NC and ZIF-8/ZnO/AC/NC composite.

ZIF-8 MOF is prepared using solvothermal method (Fig. 1.). ZIF-8 is synthesized by dissolving zinc nitrate hexahydrate (Zn (NO₃)₂·6 H₂O) in methanol to form a clear solution. And then, 2-methylimidazole (2-MIM) is added under continuous stirring, initiating the formation of ZIF-8 crystals. The resultant mixture was placed in the autoclave chamber and thereafter exposed to elevated temperatures (100 °C) in an oven for 16 h. After reaction, the product is centrifuged, washed multiple times with methanol to eliminate residual salts, and dried at 60 °C for 12 h to yield final ZIF-8 powder^28^.

### Synthesis of ZnO nanoparticles

ZnO nanoparticles are fabricated by co-precipitation method (Fig. 1.). ZnO nanoparticles are prepared by dissolving zinc acetate dehydrate (Zn (CH₃COO) ₂·2 H₂O) in ethanol and few drops of sodium hydroxide solution (NaOH) is slowly added for the precipitation of Zn (OH) ₂. The solution is stirred at room temperature for I hour to complete precipitation. The Zn (OH) _2_ is then calcined at 500 °C for 2 h to obtain ZnO nanoparticles. After cooling, the ZnO is filtered and washed several times with ethanol and then dried at 60 °C for 12 h to obtain the obtained ZnO powder^29^.

### Synthesis of activated carbon (AC)

The AC is prepared from the coconut shells (Fig. 1.); the coconut shells were collected from a local market in Cairo, Egypt (30.0444° N, 31.2357° E). First, coconut shells are cleaned properly and then dried at 60 °C for 24 h to eliminate moisture. The dried shells are then ground into small pieces to increase their surface area. The material is carbonized in an oven at 800 °C in an inert N₂ environment for 2 h to obtain biochar. The biochar is activated by treating it with KOH solution then subjecting to a second heating at 800 °C for 2 h. The activated carbon is washed with ethanol after activation to remove excess of reactants and subsequently dried at 60 °C overnight.

### Synthesis of nanocellulose (NC)

NC can be prepared from rice straw (Fig. 1.) by shredding and cleaning it, followed by NaOH treatment for the elimination of lignin and non-cellulosic fabrication. After washing, the material is treated with H₂SO₄ for further purification. Then the cellulose is mechanically treated using high-pressure homogenization to form nanofibers. After washing and drying at 60 °C for 24 h, the final nanocellulose product is obtained.

### Synthesis of ZIF-8/ZnO-activated carbon/nanocellulose composite (ZIF-8/ZnO/AC/NC)

The ZIF-8/ZnO/AC/NC composite is fabricated by mixing the synthesized ZIF-8, ZnO NPs, AC and NC in a proportion of 2:1:1:1 (Fig. 1). Amount of 2.5 g ZIF-8, 1.25 g ZnO NPs, 1.25 g AC and NC, are weighed out constantly. The ingredients were then dispersed into 100 mL of ethanol, and stirred slightly to assist solubilization. The solution is then 60 min sonicated to have a homogeneous dispersion. The solution is magnetically stirred at room temperature for 1 h after sonication. Finally, the solvent is evacuated by drying the mixture at 60 °C for 12 h and a brown ZIF-8/ZnO/AC/NC powder is obtained.

### Preparation of dye solutions

Stock solutions of Methylene Blue (MB) and Methyl Orange (MO) at a concentration of 100 mg/L were produced by dissolving accurately quantified amounts of each dye in deionized (DI) water. Operational solutions were prepared by diluting the stock to designated concentrations (50, 100, 150, 200, and 250 mg/L) prior to each experiment. The pH of the dye solutions was initially modified using 0.1 M HCl or 0.1 M NaOH and was measured with a calibrated pH meter.

### Photocatalytic degradation setup

**Fig. 2:**
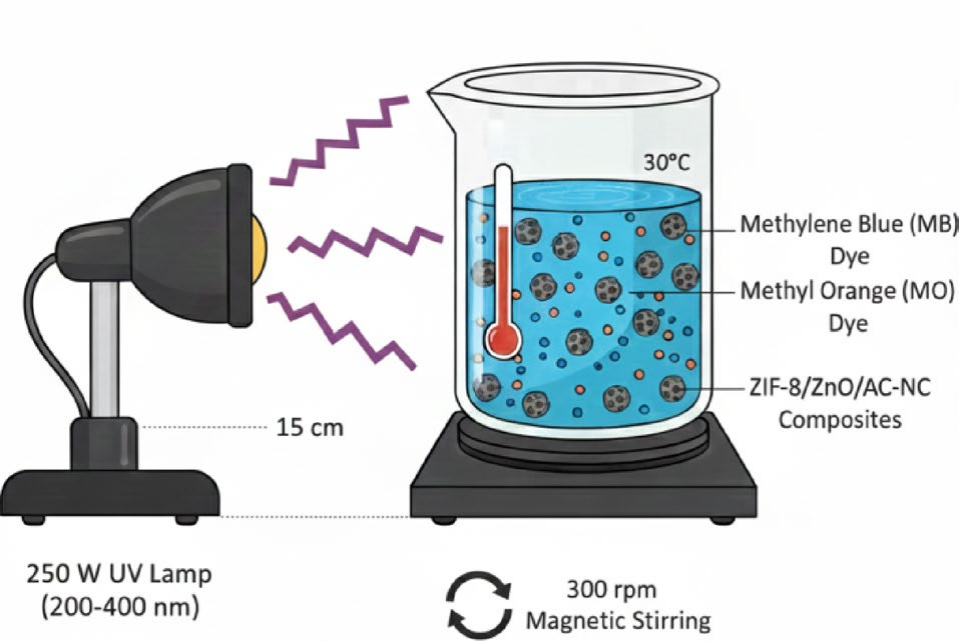
Schematic representation of the batch photoreactor system employed for UV-driven photocatalytic degradation of MB and MO utilizing the ZIF-8/ZnO/AC/NC composite.

Photocatalytic degradation of dyes was performed in an appropriately optimized experimental apparatus to assess the efficiency of performance for ZIF-8/ZnO/AC/NC Composites UV light irradiation at controlled temperature (30 °C) (Fig. 2.). It was designed to resemble real-world scenario where UV light can be simulated source for photocatalytic reaction.

A cylindrical glass reactor was employed to hold the dye solution and photocatalyst composite. For degradation, glass reactor was used and the reaction mixture was kept in closed state to avoid contamination and for controlled degradation. A 250 W UV lamp served as the irradiation source, emitting light in the 200–400 nm range, with a dominant emission wavelength at ~ 365 nm (UV-A region) to match the UV absorption characteristics of the composite. The space between lamp and reactor was kept the same at 15 cm to have uniform light intensity amongst all experiments.

The composite was dispersed in the dye solution while it was magnetically stirred at a fixed speed of 300 rpm to obtain a good mix and homogeneous suspension. The system was kept in the dark for 60 min to allow the dye molecules to adsorb onto the composite surface, so that degradation should be mainly due to photocatalysis rather than simple adsorption. Once the dark adsorption phase was complete, the UV lamp was switched on to initiate UV light-driven photocatalysis. The temperature was kept at 30 °C due to the thermal effects of photocatalytic activity. Samples of the solution were withdrawn at intervals (e.g., 5, 15, 30, 45 min) for analysis. The dye concentration in the solution was determined by measuring the absorbance of a UV-Vis spectrophotometer at characteristic MB (664 nm) and MO (464 nm) wavelengths.

### Reusability and stability

For the evaluation of stability and reusability of the composite, upon each cycle of dye degradation, the photocatalyst was recovered from the dye solution by centrifugation, washed with deionized water to remove any adsorbed dye. The photocatalyst was then reused in subsequent cycles to evaluate its performance over multiple degradation runs. The degradation efficiency in subsequent cycles was measured to determine any decline in catalytic activity due to potential material deactivation or leaching.

### Determination of point of zero charge (pHZPC) and its role in pH-dependent photocatalytic degradation

The surface charge characteristics of photocatalysts significantly influence adsorption behavior and photocatalytic degradation efficiency, particularly for ionic dye molecules. Therefore, the point of zero charge (pHZPC) of the ZIF-8/ZnO/AC/NC composite was determined using the pH drift method to clarify the role of electrostatic interactions during MB and MO degradation. In a typical experiment, 50 mL of 0.01 M NaCl electrolyte solution was transferred into a series of sealed conical flasks. The initial pH (pHi) of each solution was adjusted within the range of 2–12 using 0.1 M HCl or 0.1 M NaOH solutions. Subsequently, 0.05 g of the photocatalyst composite was added to each flask and magnetically stirred at 300 rpm for 24 h at 25 ± 1 °C to ensure equilibrium between the solid surface and electrolyte solution. After equilibration, the final pH (pHf) values were measured using a calibrated digital pH meter. The difference between initial and final pH values (ΔpH = pHf − pHi) was plotted against pHi to determine the intersection point where ΔpH equals zero, corresponding to the pHZPC.

### Characterization tools

The synthesized nanocomposites were characterized using several analytical techniques to assess their structural, morphological, and chemical properties. The material’s morphology was examined using SEM (JEOL JSM-7600 F). The analysis of chemical bonds and functional groups in the nanocomposite was conducted using FTIR (Thermo Scientific Nicolet iS10), with spectra acquired in the range of 4000–500 cm⁻¹. The crystalline phases were examined via X-ray diffraction (XRD) using a Bruker D8 Advance with Cu Kα radiation (λ = 1.5406 Å) throughout a 2θ range of 10° to 70°. The identification and valences of the present chemical elements in the supplied samples were evaluated using X-ray Photoelectron Spectroscopy (XPS). The measurements were performed with a VG MultiLab 2000 photoelectron spectrophotometer at ambient temperature, within a vacuum range of 10^−8 to 10^−9 Torr, employing Mg Kα radiation, at power settings of 10 keV and 20 mA. The surface area and porosity of the nanocomposite were assessed using a BET analyser (Micromeritics ASAP 2020), with nitrogen adsorption-desorption isotherms acquired at 77 K. Photoluminescence (PL) spectra were acquired using a Horiba Scientific FluoroMax-4 spectrofluorometer to assess the optical characteristics of the nanocomposites. The UV-Vis absorption spectra were obtained using a Shimadzu UV-2600 spectrophotometer to analyze the band gap and other electronic characteristics of the material.

## Results and discussion

### catalysts characterization

**Fig. 3:**
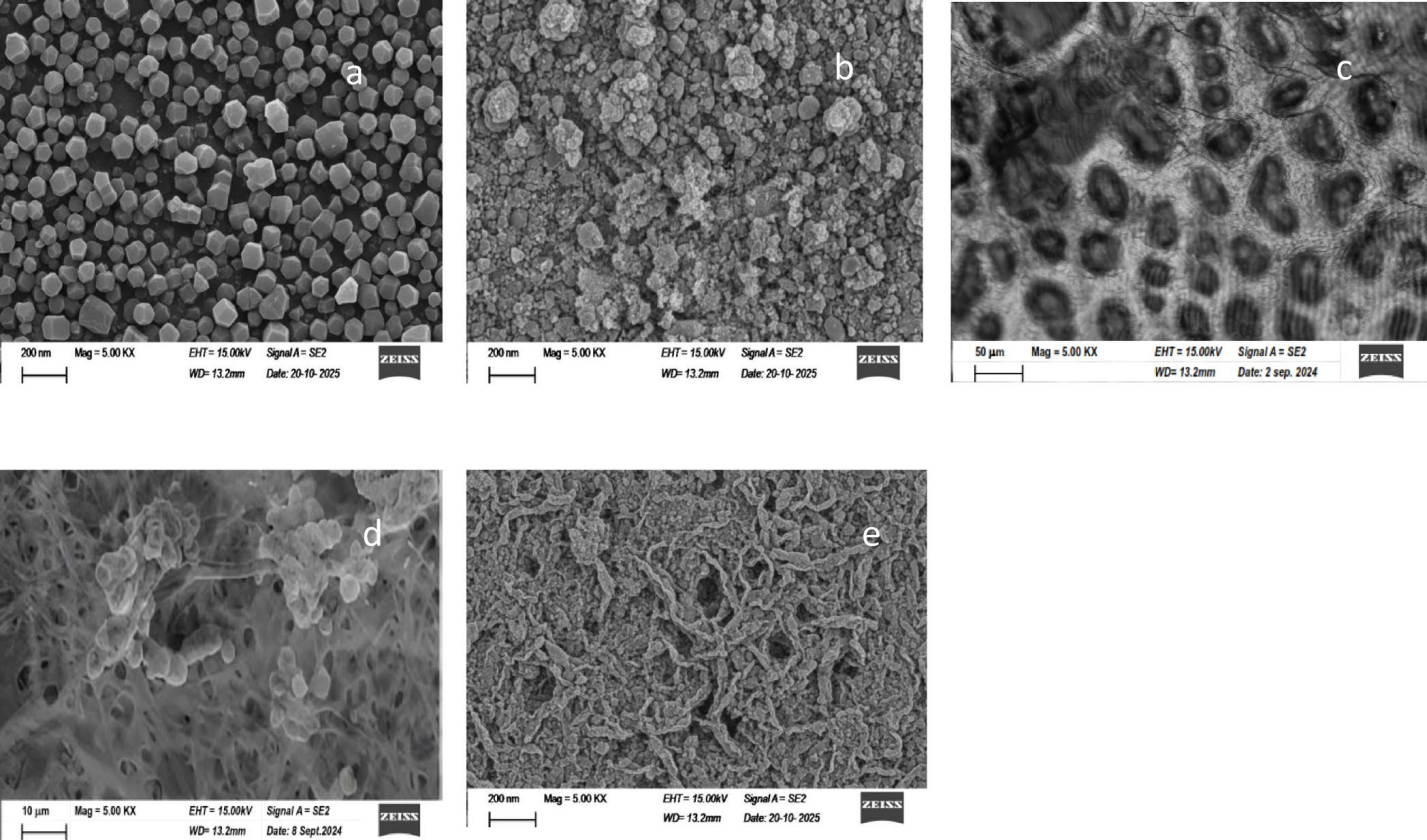
SEM images of (**a**) ZIF-8, (**b**) ZnO, (**c**) AC, (**d**) NC and (**e**) ZIF-8/ZnO/AC/NC composites.

Figure 3 represents SEM of ZIF-8, ZnO, AC, NC and ZIF-8/ZnO/AC/NC composite. The SEM image of ZIF-8 (Fig. 3a) shows well-defined hexagonal crystals with a uniform size distribution. The particles have sharp edges and flat faces, which is typical of ZIF-8’s crystalline structure. This shape contributes to the material’s high surface area and porosity. While the SEM image of ZnO (Fig. 3b) shows a rough, irregular surface with particles that appear to cluster together. The particles are not uniform in shape, giving the material a textured appearance.

The SEM image shows AC particles (Fig. 3c) that have an irregular, mostly circular or oval shape. The particles appear rough and uneven, with noticeable indentations and cavities across their surfaces. Meanwhile, the SEM image reveals the NC particles (Fig. 3d) to have a fibrous and interconnected structure. The particles appear as fine, elongated fibers, forming a network-like arrangement. Some clusters of particles are visible, indicating the formation of aggregates, while the overall structure shows a highly porous and fibrous morphology at the nanoscale.

The SEM image of the ZIF-8/ZnO/AC/NC composite (Fig. 3e) reveals a unique and intricate morphology, showcasing a complex structure with fine details. The composite material appears to exhibit a rough, interconnected network of nanosheets with an irregular and porous surface. The morphology likely reflects the interaction between ZIF-8, ZnO, AC, and NC, contributing to enhanced surface area and porosity.

**Fig. 4:**
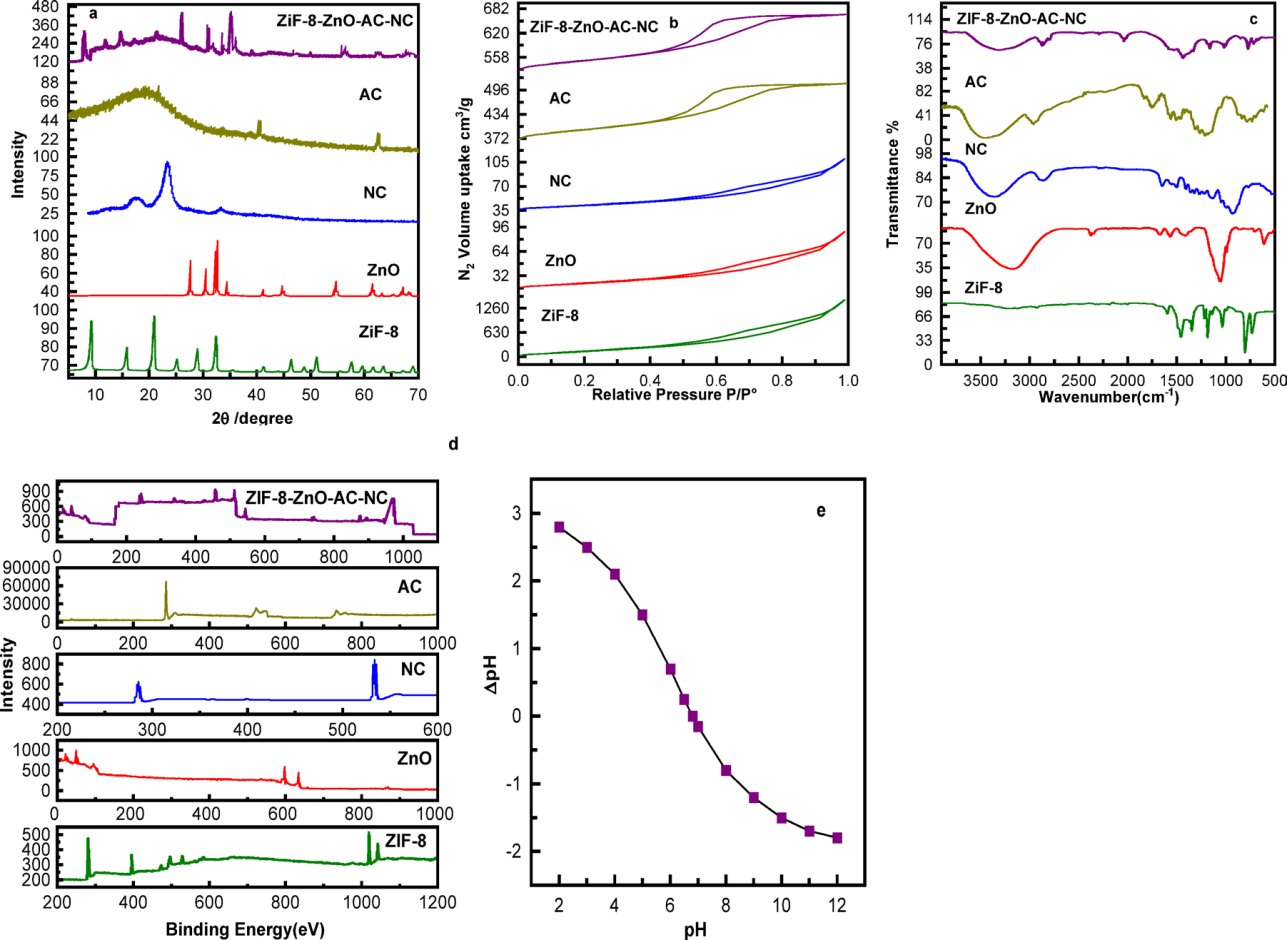
(**a**) XRD pattern, (**b**) Nitrogen adsorption-desorption isotherm, (**c**) FTIR spectra and (**d**) XPS of ZIF-8, ZnO, NC, AC and ZIF-8/ZnO/AC/NC composites, (**e**) pHZPC of ZIF-8/ZnO/AC/NC.

The crystalline structure of the fabricated materials was evaluated by XRD (Fig. 4a). The ZIF-8 material exhibited several intense peaks at 7.3°, 10.4°, 12.7°, and 18.0°, which are characteristic for its sodalite structure^30^. The sharp peaks at 2θ values of 31.8°, 34.4°, 36.3°, 47.6°, 56.6°, and 62.9° correspond to the (100), (002), (101), (102), (110), and (103) planes of ZnO (JCPDS card No. 89–0511), respectively^31^. The broad peak at a 2θ value of approximately 22° can be attributed to the amorphous nature of the NC^32^. No distinct peaks associated with AC were observed, which is consistent with its typically amorphous or highly disordered structure. The XRD pattern of the composite material does not display distinct new peaks, but rather reflects the combined characteristics of ZIF-8, ZnO, AC and NC. The specific surface area and pore structure of ZIF-8, ZnO, NC, AC and ZIF-8/ZnO/AC/NC composites were analyzed using nitrogen adsorption-desorption isotherms in conjunction with the Brunauer–Emmett–Teller (BET) theory (Table 1) and (Fig.4b). 

**Table 1:** Surface area of prepared samples

Materials	Surface area (m²/g)	Pore size (nm)	Pore Volume (cm^3^/g)
ZIF-8	1510.3	1.1	0.63
ZnO	70.7	5.3	0.21
NC	100.5	6.4	0.25
AC	500	1.4	0.32
ZIF-8/ZnO/AC/NC	655.3	4.3	0.48

ZIF-8 shows a very high surface area of 1510.3 m²/g with a micropore size of 1.1 nm, while ZnO and NC exhibit mesopore sizes of 5.3 nm and 6.4 nm with surface areas of 70.7 m²/g and 100.5 m²/g, and pore volumes of 0.21 and 0.25 cm³/g, respectively. AC provides a high surface area of 500 m²/g with 1.4 nm pores and a pore volume of 0.32 cm³/g. The ZIF-8/ZnO/AC/NC composite exhibited a surface area of 655.3 m²/g, a pore size of 4.3 nm and a pore volume of 0.48 cm³/g, confirming the formation of a hierarchical micro–microporous structure. Notably, the enhanced surface area, combined with the hierarchical pore structure and significant pore volume, facilitates greater reactant adsorption and improved mass transfer, which contributes to the superior catalytic performance of the composite.

Figure 4c presents the FTIR spectra for the ZIF-8, ZnO, NC, AC and ZIF-8/ZnO/AC/NC composites. The FTIR spectra of ZIF-displays the principal vibrational characteristics anticipated for the ZIF-8 structure. The faint bands at 3138 and 2933 cm^− 1^ correspond to the aromatic and aliphatic C–H stretches, respectively, of the imidazole group. The peak at 1585 cm^− 1^ is ascribed to C = N stretching, whereas the complex bands between 1350 and 1500 cm^− 1^ result from imidazole ring stretching^33^. The bands within the range of 900–1350 cm^− 1^ correspond to in-plane bending of the imidazole ring, whereas those below 850 cm^− 1^ are linked to out-of-plane bending. A prominent band at 420 cm^− 1^ is associated with Zn–N stretching^34^.The spectrum of ZnO nanoparticles exhibits a peak near 560 cm^− 1^, indicating the existence of Zn-O bonds, and the broad absorption peak at 3438 cm-1 is attributable to the distinctive absorption of hydroxyl groups^35,36^.

The spectrum of the NC exhibits a broad and intense peak around 3300 cm^− 1^, which is attributed to the O-H stretching vibration of hydroxyl groups^37^. The peaks observed at 2911 cm^− 1^ and 1420 cm^− 1^ correspond to C-H stretching and bending vibrations, respectively^38^. While, the FTIR spectrum of AC displays a broad peak at 3400 cm^− 1^, indicative of O-H stretching, and a peak at 1720 cm^− 1^, which corresponds to C = O stretching in carboxyl groups^39–41^. The FTIR spectrum of the composite exhibits distinct peaks corresponding to ZIF-8, ZnO, NC, AC, signifying the successful integration and interaction of these materials.

The XPS survey spectrum of ZIF-8 shows the typical elemental composition expected for this metal–organic framework (Fig. 4d). A dominant peak of C 1 s can be observed at 285 eV due to the organic 2-methylimidazole linker. The N 1 s peak was also observed around 399–401 eV, which indicated the existence of Zn²⁺ coordinated by nitrogen in the imidazolate rings. Moreover, the spectrum exhibits the typical Zn 2p peaks at ~ 1021.7 and 1044.8 eV ascribable to the presence of Zn^2+^ species present in the framework of ZIF-8. The overall profile does not show any additional or unexpected peaks, indicating that the sample is chemically pure and that the Zn–N coordination environment of ZIF-8 has been successfully formed. These features agree well with previously reported XPS data for ZIF-8 in the literature^17^.While, the XPS survey spectrum of ZnO (Fig. 4d) shows the characteristic peaks corresponding to Zn and O. A prominent peak of O 1 s appears at 530.6 eV, which is typical for lattice oxygen (O²⁻) in ZnO. Zn 2P peaks in the highs binding energy region was clearly detected, with their corresponding peak at about of 1021.41 eV and 1044.69 eV, respectively, confirming the presence of Zn²⁺^42^.

The XPS spectrum of NC (Fig. 4d) reveals key carbon and oxygen features. The strong carbon 1 s peak at ∼285 eV reflects the C–C and C–H bonds in the cellulose backbone. The oxygen 1 s peak at 532–533 eV is assigned to the hydroxyl (–OH) and ether (–O–C) groups, which are typically found on the surface of NC^43,44^.

The XPS spectrum of AC reveals key features of its surface chemistry. The 284.2 eV peak is attributable to C 1 s and represents carbon in a sp² hybridized form, known as the carbon skeleton in activated carbon. The peak at around 530.6 eV is attributed to O 1 s, indicating the presence of oxygen-containing groups (e.g., -OH, C = O and -COOH) on the surface^45^.

The XPS spectra of the ZIF-8/ZnO/AC/NC composite show the presence of its individual components. The presence of carbon, oxygen, zinc, and nitrogen signals indicates that activated carbon, zinc oxide, nitrogen doping, and ZIF-8 are effectively incorporated, preserving their characteristic surface properties in the composite.

The obtained results indicate that the pHZPC value of the composite is approximately 6.8 (Fig. 4e) and Table 2. Below this pH value, the catalyst surface becomes positively charged due to protonation of functional groups originating from ZIF-8 imidazole nitrogen sites, nanocellulose hydroxyl groups, and oxygenated functionalities of activated carbon. Conversely, at pH values above 6.8, surface deprotonation generates negatively charged active sites.

This surface charge transition is a key factor in the photocatalytic degradation performance. Methylene blue (MB) is a cationic dye, thus under the alkaline environment where catalyst surface becomes negatively charged the electrostatic attraction with increased strength would facilitate adsorption and subsequent radical oxidation. On the other hand, since MO is a dye with anionic structure, more it interacts in acidic environments where surface of the catalyst is positive-charged.

Therefore, the pHZPC results offer mechanistic evidence for the observed maximum degradation efficiencies taking place near neutral pH where adsorption equilibrium, radical generation and charge separation all work together to improve photocatalytic behavior.

**Table 2:** Relationship between surface charge and dye interaction.

pH range	Surface charge	MB interaction	MO interaction
pH < 6.8	Positive	Repulsion	Strong attraction
pH ≈ 7	Neutral	Balanced	Balanced
pH > 6.8	Negative	Strong Attraction	Repulsion

**Fig. 5:**
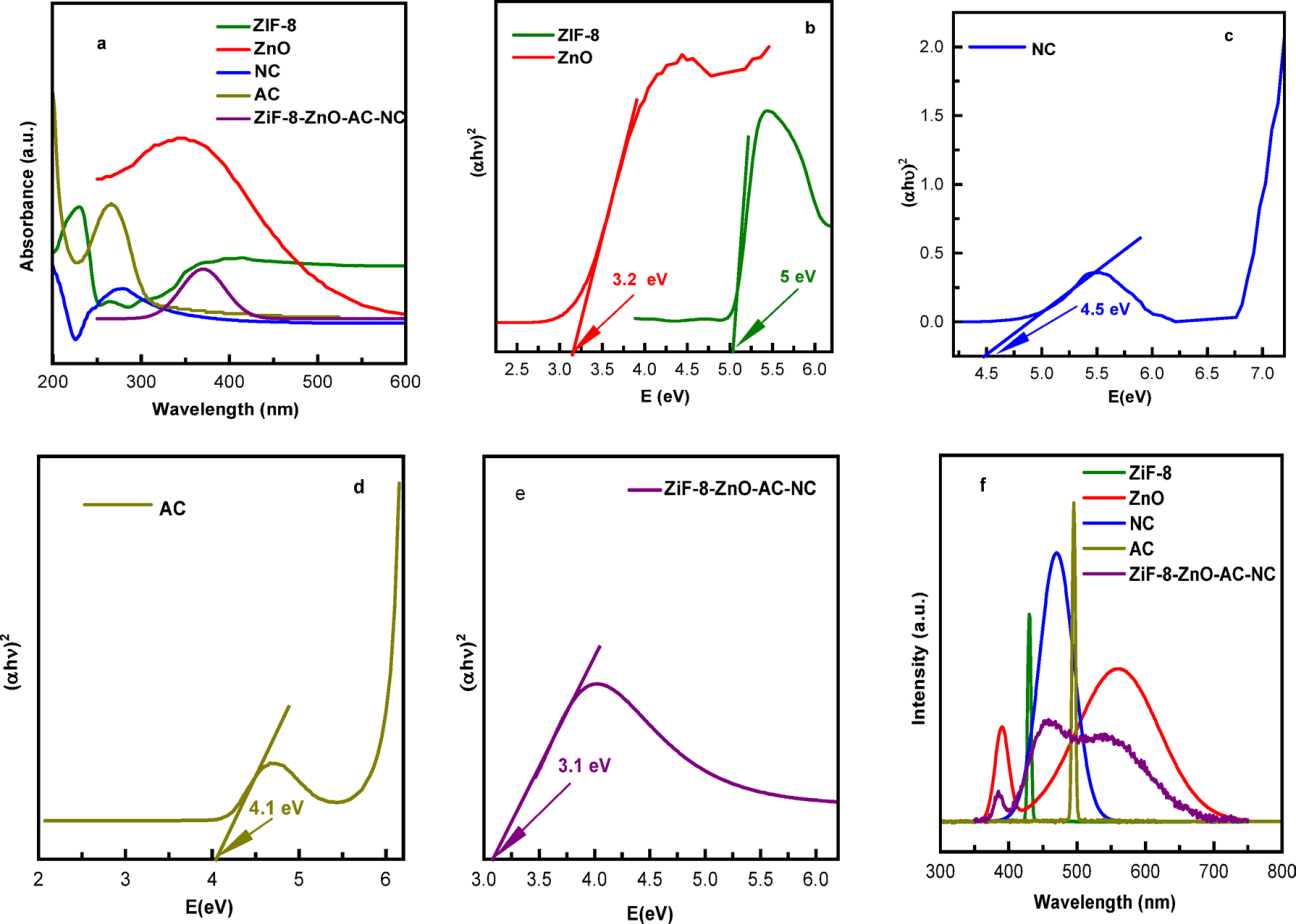
(**a**)Uv-Vis, (**b**,**c**, d and **e**) Band gap, and (**F**) PL spectra of ZIF-8, ZnO, NC, AC and ZIF-8/ZnO/AC/NC composites.

The UV-Vis absorption plots of ZIF-8, ZnO, NC and AC and ZIF-8/ZnO/AC/NC composites exhibited distinct features (Fig. 5a). ZIF-8 shows a maximum absorption peak at 230 nm, indicating a considerable absorption in the UV range owing to its metal organic framework nature^46^. ZnO has a strong absorption at 388 nm associated with its wide bandgap which can effectively absorb UV. NC had a wider absorption edge that is from ∼250 nm to 500 nm with the maximum peak of around 275 nm.AC shows absorbance peak at 302 nm. Finally, the ZIF-8/ZnO/AC/NC composite also integrates the characteristics of each component, with peaks around 387 nm, suggesting improved light absorption for photocatalysis applications.

The band gap analysis exhibits different characteristics for ZIF-8, ZnO, NC and AC and ZIF-8/ZnO/AC/NC composites (Fig. 7,b, c,d, and e)^47^. ZIF-8 possesses a band gap of about 5 eV, indicating its ability to absorb light primarily in the UV range, which is typical for metal-organic frameworks^46^. ZnO has a band gap of ∼3.2 eV, which makes it an efficient absorber for UV radiation. While, NC has a 4.5 eV band gap, indicating its good UV absorber^48^.while, The band gap of AC is 4.1 eV. Finally, the band gap of ZIF-8/ZnO/AC/NC (3.1 eV) is much smaller than that of the individual components suggesting better light absorption throughout UV region and enhanced photocatalytic performance^49,50^. This composite material, with its lower band gap, demonstrates a broader spectrum of light absorption, making it ideal for photocatalytic and light-driven applications.

The PL spectra of ZIF-8, ZnO, NC, and AC and ZIF-8/ZnO/AC/NC composites exhibit distinct emission features for each component (Fig. 7f). ZIF-8 exhibits a sharp blue emission peak around 430–450 nm, which can be attributed to linker-centered transitions.

The typical broad visible emission due to ZnO is observed in the region between 520 and 560 nm which are linked to the presence of defect states such as oxygen vacancies. NC exhibits a medium blue emission at around 470–490 nm, whereas AC possesses a very weak and broad PL band over the whole range from 400 to 600 nm ascribed probably to its high conjugation.

In the ZIF-8–ZnO–AC–NC composite, the PL curve becomes broader, spanning roughly 380–700 nm, with a main visible emission region near 520–600 nm and smaller shoulders in the 430–480 nm range. Notably, the overall PL intensity of the composite is significantly reduced compared with the individual components, especially ZnO and ZIF-8. This decrease in PL intensity indicates a lower recombination rate of photogenerated electron–hole pairs. Since PL emission results from the radiative recombination of excited charge carriers, a weaker PL signal suggests more efficient charge separation and longer carrier lifetimes. This allows a greater number of electrons and holes to participate in photocatalytic surface reactions rather than recombining^47^.

The improved charge separation in the composite can be attributed to the formation of heterojunctions between ZnO and ZIF-8, as well as the presence of activated carbon and nanocellulose, which facilitate electron transport^51^. The carbon components act as electron acceptors and conductive pathways, promoting charge migration and suppressing recombination^52^. As a result, the composite exhibits enhanced charge carrier separation efficiency compared with the individual materials.

Based on the PL intensity results, the recombination rate follows the order: AC > NC > ZIF-8 > ZnO > ZIF-8/ZnO/AC/NC, while the photocatalytic activity follows the opposite trend. The lowest PL intensity observed for the composite confirms its superior charge separation efficiency, which explains its enhanced photocatalytic performance. This relationship between reduced PL intensity and improved photocatalytic activity has been widely reported for semiconductor-based photocatalysts^53^.

### Photocatalytic activity

**Fig. 6:**
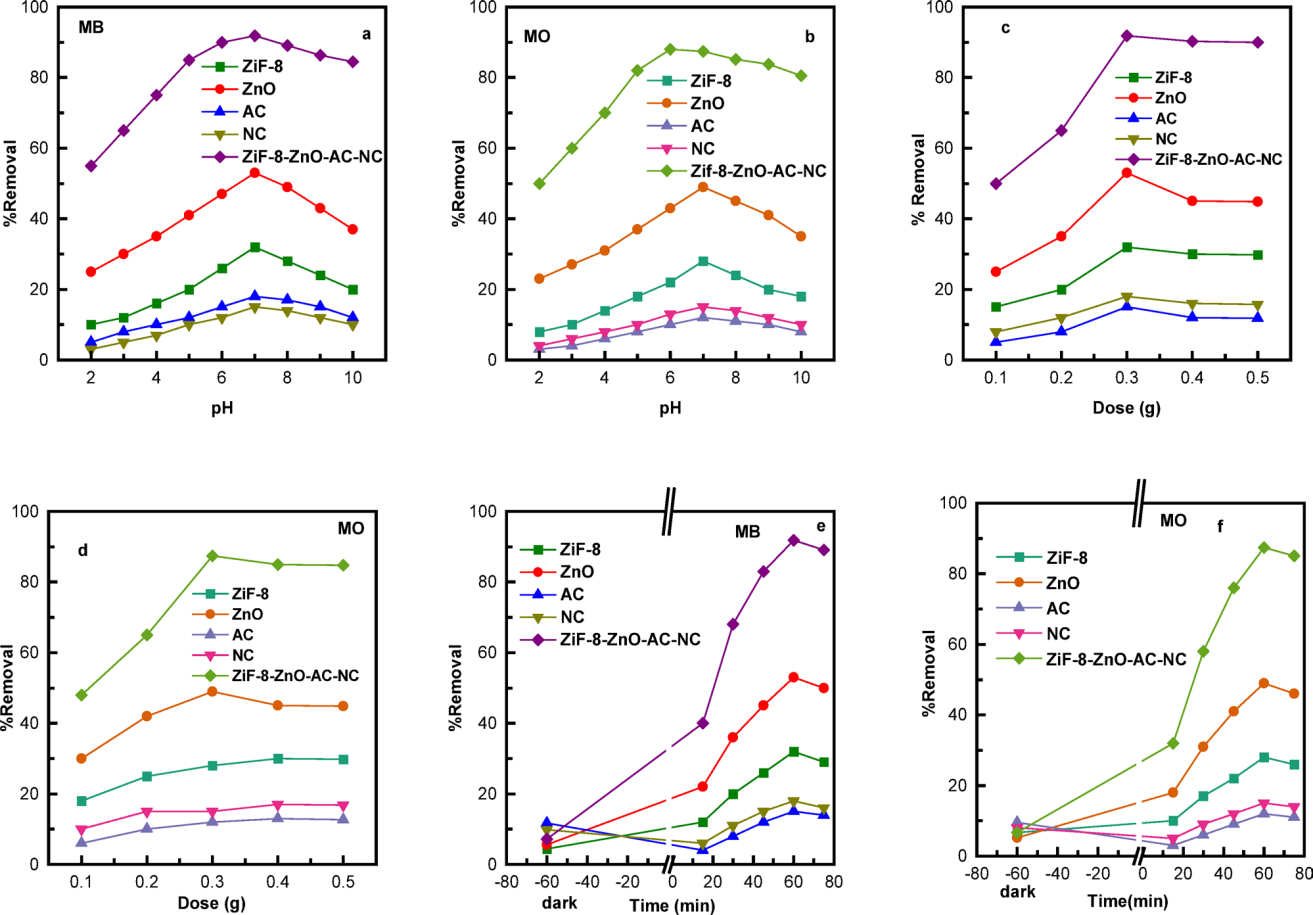
Effect of (**a**) and (**b**) pH, (**c**) and (**d**) dose, and (**e**) and (**f**) reaction time on the photocatalytic degradation of MB and MO using ZIF-8, ZnO, NC, AC and ZIF-8/ZnO/AC/NC composites.

The impact of pH on the photocatalytic degradation of MB and MO was investigated with the ZIF-8/ZnO/AC/NC composite and its components: ZnO, ZIF-8, NC, and AC (Fig. 6a). Results show that degradation efficiency increases as the pH rises from 2 to 7, reaches maximum at pH value of 7, followed by decrement when increasing the pH (8–10). This trend is the same for both dyes, and the ZIF-8/ZnO/AC/NC composite exhibits best adsorption compared to its constituents at pH 7.

For MB, ZnO/ZIF-8-NHACN composite resulted in 91.8% degradation in pH 7 while for the case of ZnO only is 53%, and for ZIF-8 was 32%. Comparison of the degradation efficiencies NC and AC exhibited significantly lower efficiencies being 26% and 15% at pH 7, respectively. Best degradation at pH 7 can be attributed to the surface charge and dye adsorption ability of ZnO, which are favorable for a neutral environment^54,55^. When the pH further exceeds 7, even degradation efficiency decreases and this might be attributed to that lower adsorption and as well as interactions between the catalyst surface and the dye molecules at more alkaline pH.

The same behavior was obtained for MO (Fig. 6b), the ZIF-8/ZnO/AC/NC composite showed 87.4% of activity at pH 7 compared to 49% by ZnO alone, 28% for ZIF-8 and negligible for AC and NC. The higher degradation as the pH increased from 2 to 7 is due to the most excellent adsorption of negatively charge MO molecules on surface of ZnO which would have enhanced photocatalytic activity^56^. The degradation performance exhibited a lower efficiency at pH 8 and pH 10, which was attributed to the decline of the surface charge weakening as well as lower adsorption efficiency on both higher pH.

The influence of catalyst dose on the photocatalytic degradation MB and MO was studied using a composite material (ZIF-8/ZnO/AC/NC) and their constituents (ZnO, ZIF-8, NC, AC) for comparison (Fig. 6c and d). The 0.3 g/L ZIF-8/ZnO/AC/NC was the most efficient for the decomposition of the dyes MB (91.8%) and MO (87.4%), indicating its excellent photocatalytic performance. The individual components, while effective to some degree, showed lower degradation efficiencies: ZnO achieved 53% for MB and 49% for MO, and the other materials (ZIF-8, NC, and AC) had even lower values, with AC showing the least effectiveness at 15% for MB and 12% for MO. When the amount of catalyst was higher than 0.3 g/L, the degradation rates for each material including composite decreased. This decrease may be due to light scattering, particle aggregation and the saturation of adsorption which retard the photocatalytic reaction at a higher concentration of the catalysts^57^. Thus, the optimum dose for photocatalytic degradation of both dyes was determined to be 0.3 g/L as it gives maximum efficiency of the composite material.

The adsorption of MB and MO increased with time in both the dark and under UV illumination (Fig. 6e and f). Under dark conditions, limited adsorption was observed due to the interaction of dye molecules with the surface of the materials. For MB, the adsorption ratio of AC (11.7%) and NC (9.8%) was the highest at 1 h due to their high surface area and large amount of functional groups which can efficiently take up dye compared to composite, ZnO, and ZIF-8 (7.2%, 5.6%, and 4.4%, respectively). In the case of MO, a small reduction in dark adsorption was observed: 9.5% on AC and 8.1% on NC after 1 h, while it was lower for the composite (6.7%).

Under UV illumination, The composite MB degradation sharply rose, being as 91.8% after 60 min and slightly reduce to 89.1% after 75 min, while ZnO, ZIF-8 obtained the moderate removal (53%, 32%) and, and NC and AC contributed minimally. The higher activity of the composite is attributed to the synergistic effect of ZIF-8, ZnO, NC and AC that could favor charge separation, more exposure to active sites and better light adsorption^58^. Similarly, MO degradation followed the same trend, but the extent of removal was lower: the composite achieved 87.4% after 60 min, slightly decreasing to 85.1% at 75 min, whereas ZnO and ZIF-8 reached 49% and 28%, and NC and AC were less active. The relatively lower removal of MO may result from electrostatic repulsion between the anionic MO and negatively charged surfaces at neutral pH, while the cationic MB interacts more favorably with the materials. Overall, the enhanced removal under UV illumination is primarily attributed to the progressive generation of reactive species (•OH, O₂ •⁻) which chemically degrade the dye molecules, while slight decreases after 60 min may be due to active site saturation, or charge carrier recombination^58^.

**Fig. 7:**
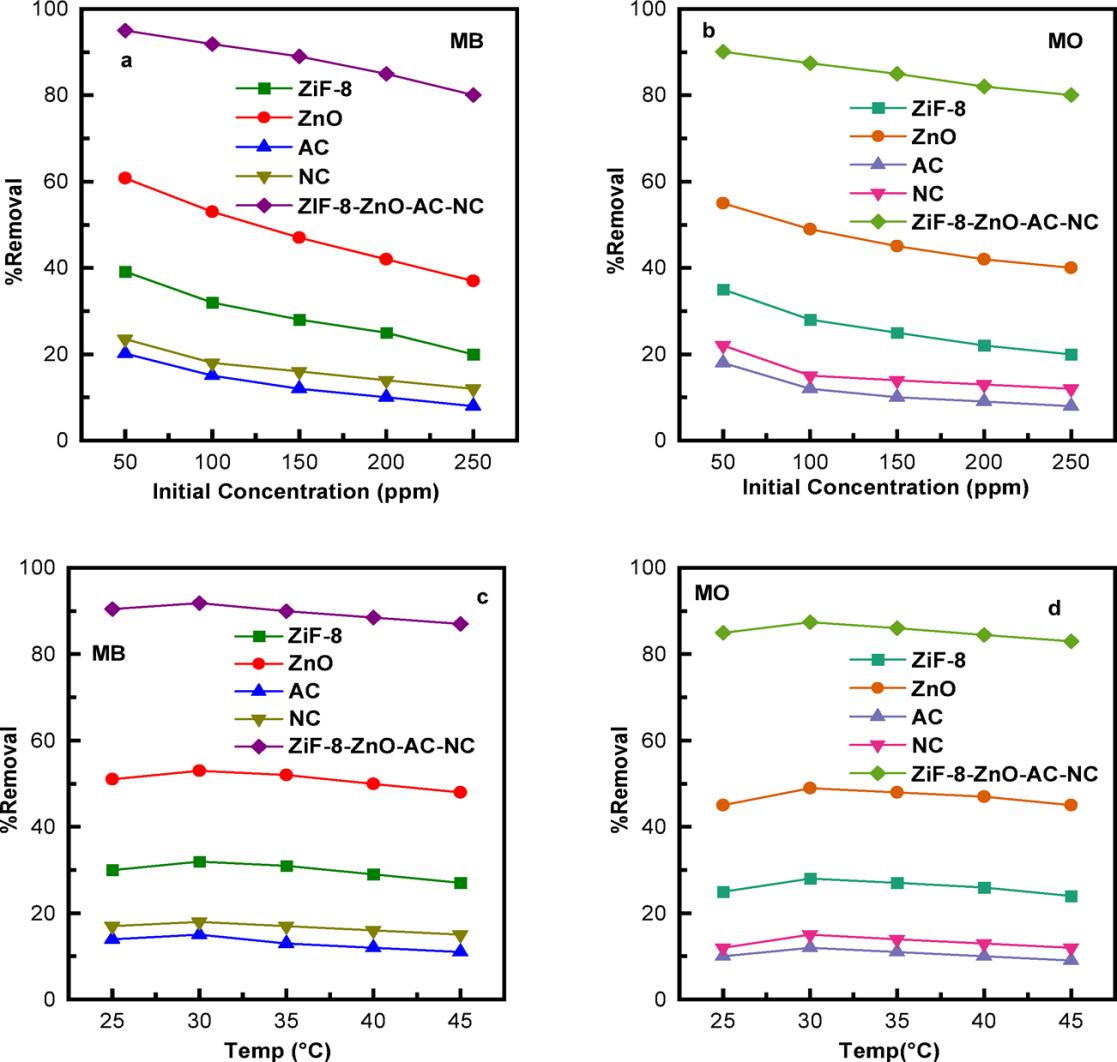
Effect of (**a**) and (**b**) Initial Concentration, and (**c**) and (**d**) Temperature on the photocatalytic degradation of MB and MO using ZIF-8, ZnO, NC, AC and ZIF-8/ZnO/AC/NC composites.

Photocatalytic MB and MO degradation efficiency is strongly influenced by the initial dye concentration (Fig. 7a, b). The degradation rate of the ZIF-8/ZnO/AC/NC composite to MB decreases from 95% to 80%, along with the concentration increasing from 50 mg/L to 250 mg/L. From a similar trend, for MO, the degradation decreases from 90.1% at 50 mg/L to 79.5% at 250 mg/L. The reduction in performance is due to factors such as saturation of active sites where more dye molecules provide less surface area for adsorption, light scattering which reduces the penetration of light energy onto the catalyst as well as competitive adsorption that leads to less number of dye molecules interacting with the catalyst^59^. Moreover, high concentrations may cause electron-hole recombination, further reducing catalytic efficiency^60^. Consequently, better degradation efficiency can be achieved at lower dye concentrations especially about 50 mg/L.

The MB and MO was measured under temperature ranging from 25 to 45 °C for the ZIF-8/ZnO/AC/NC composite and their component separately (Fig. 7c and d). For MB, the composite exhibited the best removal rate of 91.8% at 30 °C and slightly decreased with the increase in temperature (90.0% at 35 °C, 88.5% at 40 °C and 87.0% at 45 °C). ZnO and ZIF-8 exhibited low adsorption (maximum 53% and 32% at 30 °C), whereas NC and AC were less active. For MO, a similar trend was observed: the composite removed 87.4% at 30 °C, with gradual decreases at higher temperatures, whereas ZnO and ZIF-8 reached 49% and 28% degradation, respectively.

The slightly higher degradation at 30 °C may be due to the increased molecular mobility that favors dye penetration in the catalyst surface and promotes effective adsorption. Beyond 30 °C, the gradual decrease in removal is attributed to increased recombination of photo-induced e −/h + pairs, shortened lifetime of reactive species (•OH​/O2•⁻), and potentially partial desorption of dyes from the catalyst surface. Moreover, high temperatures might decrease the adsorption efficiency of NC or AC, impeding the overall photocatalytic activity. This suggests that moderate temperatures are beneficial for achieving a best compromise between adsorption process and photocatalytic reaction, in turn leading to the highest dye degradation.

### Kinetic models

**Fig. 8:**
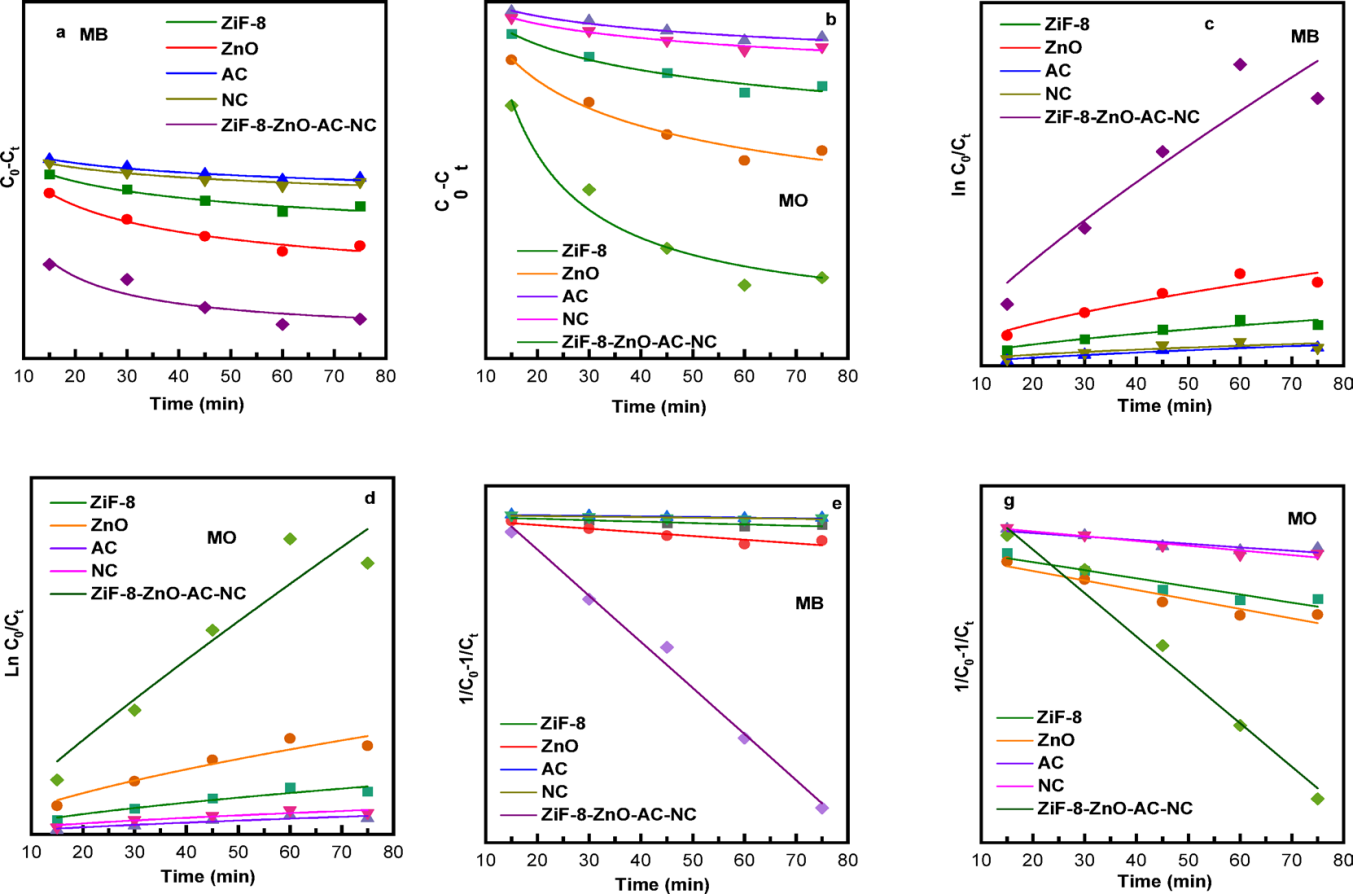
(**a**) and (**b**) Zero- pseudo order, (**c**) and (**d**) pseudo-first-order, (**e**) and (**f**) pseudo-second-order kinetic models for the photocatalytic degradation of MB and MO using ZIF-8, ZnO, NC, AC and ZIF-8/ZnO/AC/NC composites.

The kinetic analysis of MB and MO degradation with the various catalysts showed that pseudo-second order model fitted best in describing the degradation process for both dyes (Fig. 8. and Table 3). The kinetic parameters (k₀, k₁, and k₂) were calculated from the linearized forms of the respective models, and their suitability was evaluated based on the correlation coefficients (R²) (Table 4).

In the case of MB, the highest R² values were obtained by ZIF-8/ZnO/AC/NC composite (0.991), suggesting a second-order mechanism which is dependent on the concentrations of both the dye and catalyst. As well as for MO, the second-order model was also obtained to have the highest R² (0.983) in the case of ZIF-8/ZnO/AC/NC composite implying that degradation followed a similar pathway. This indicates that the degradation process is more accurately described by a second-order kinetic behavior, suggesting that surface interactions and electron-transfer reactions at the catalyst interface significantly influence the overall rate.

**Table 3:** Kinetic parameters for the photocatalytic degradation of MB and MO using ZIF-8, ZnO, NC, AC and ZIF-8/ZnO/AC/NC composites.

Dye	Catalyst	Pseudo-zero-order	pseudo-first-order	pseudo-second-order
MB	ZIF-8	0.885	0.906	0.981
	ZnO	0.902	0.921	0.989
	AC	0.891	0.913	0.981
	NC	0.899	0.917	0.987
	ZIF-8/ZnO/AC/NC	0.914	0.931	0.991
MO	ZIF-8	0.896	0.905	0.980
	ZnO	0.906	0.907	0.982
	AC	0.894	0.897	0.975
	NC	0.877	0.875	0.979
	ZIF-8/ZnO/AC/NC	0.907	0.908	0.983

In comparison, the R² value for the pseudo-first-order and pseudo-zero order models was relatively low indicating that the degradation is mainly governed by adsorption of dye molecules on to catalyst surface followed by suitable chemical reaction^61^. In general, the second-order model was well-fitted with respect to the dye degradation process and ZIF-8/ZnO/AC/NC composite showed the greatest dye removal in both systems. The lower R² values observed for pseudo-zero-order and pseudo-first-order models suggest that simple concentration-dependent kinetics cannot adequately describe the experimental results.

**Table 4:** Rate constants for pseudo-zero-order (k₀), pseudo-first-order (k₁), and pseudo-second-order (k₂) kinetics for the photocatalytic degradation of MB and MO using ZIF-8, ZnO, AC, NC, and ZIF-8/ZnO/AC/NC composites.

Dye	Catalyst	k₀ (mg L⁻¹ min⁻¹)	k₁ (min⁻¹)	K₂ (g mg⁻¹ min⁻¹)
MB	ZIF-8	0.307	0.0165	0.42
	ZnO	0.487	0.0172	0.085
	AC	0.247	0.0219	0.21
	NC	0.267	0.0162	0.33
	ZIF-8/ZnO/AC/NC	0.547	0.0248	0.012
MO	ZIF-8	0.287	0.0187	0.000181
	ZnO	0.493	0.0190	0.000212
	AC	0.147	0.0225	0.000080
	NC	0.160	0.0182	0.000105
	ZIF-8/ZnO/AC/NC	0.904	0.0272	0.001021

### Isotherm models

**Fig. 9:**
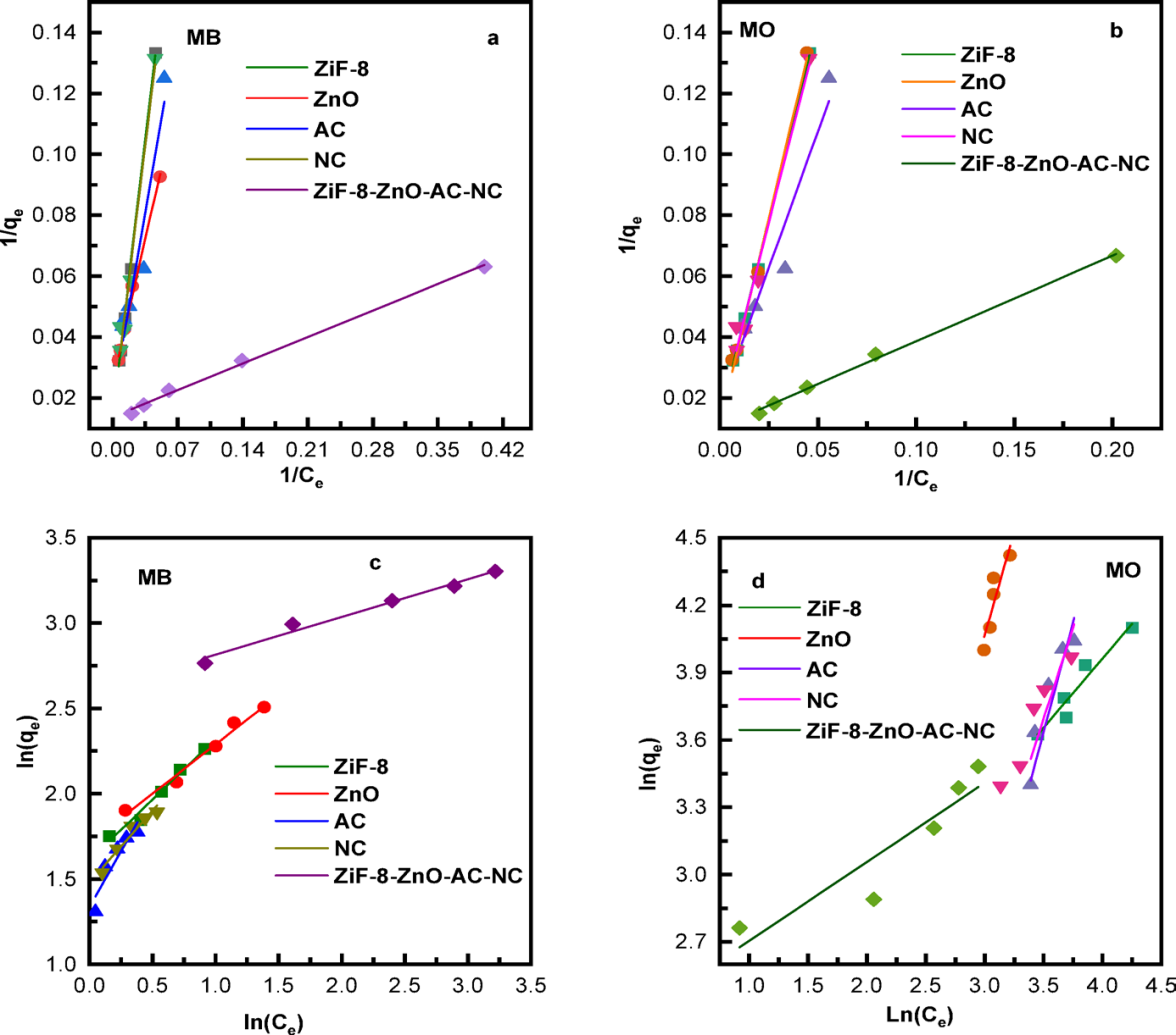
(**a**) and (**b**) Langmuir and (**c**) and (**d**) Freundlich models for the photocatalytic degradation of MB and MO using ZIF-8, ZnO, NC, AC and ZIF-8/ZnO/AC/NC composites.

The adsorption isotherm values of MB and MO dyes demonstrate a clear and consistent trend among the individual components and the composite catalyst (Fig. 9; Table 5). For both dyes, the ZIF-8/ZnO/AC/NC composites demonstrate the superior adsorptive efficiency with extremely high Langmuir qmax (53.0 mg/g for MB and 50.1 mg/g for MO) and excellent linearity (R² ≈ 0.998–0.997), confirming highly favorable monolayer adsorption. The individual components also show the same trend with both dyes, with a closely similar order of performance.

ZnO and ZIF-8 show a moderate adsorption capability corresponding relatively well to both Langmuir and Freundlich models, whereas AC and NC always have lower adsorption capability. This is also confirmed by Freundlich constants: the composite shows the largest Kf with *n* > 1, suggesting strong and favorable heterogeneous adsorption while AC and NC show lower Kf. Overall, both MB and MO follow the same adsorption trend—composite > ZnO ≈ ZIF-8 > NC > AC demonstrating that the individual materials retain the same relative behavior toward both dyes, while the composite significantly enhances overall adsorption efficiency.

### Thermodynamic studies

The thermodynamic study of the photocatalytic degradation of MB and MO dyes (Table 6) demonstrates that this process is spontaneous in all the catalysts tested, and that a composite (ZIF-8/ZnO/AC/NC), had more favorable thermodynamics condition for MB degradation. The ΔG°of the degradation reactions is negative for all catalysts, which implies spontaneity, and the composite catalyst yielded the most favorable value towards MB degradation (ΔG° = −6.088 kJ/mol) and MO degradation (ΔG° = −4.8818 kJ/mol).

**Table 5:** Isotherm parameters for the photocatalytic degradation of MB and MO using ZIF-8, ZnO, NC, AC and ZIF-8/ZnO/AC/NC composites.

Dye	Catalyst	*R*^2^ (Langmuir)	Langmuir Qₘₐₓ (mg/g)	Langmuir Kᴸ (L/mg)	*R* ^2^ (Freundlich)	Freundlich Kꜰ (mg/g)	Freundlich (*n*)
MB	ZIF-8	0.991	0.09	15.0	0.977	5.01	1.40
	ZnO	0.983	0.10	22.5	0.975	5.55	1.73
	AC	0.994	0.07	15.0	0.852	3.80	0.76
	NC	0.986	0.08	10.0	0.947	4.39	1.19
	ZIF-8/ZnO/AC/NC	0.998	0.12	53.0	0.981	11.88	1.69
MO	ZIF-8	0.992	0.08	12.5	0.931	4.29	1.65
	ZnO	0.994	0.10	18.0	0.936	0.13	0.49
	AC	0.992	0.07	14.0	0.905	0.041	0.51
	NC	0.981	0.09	9.5	0.970	1.19	0.97
	ZIF-8/ZnO/AC/NC	0.997	0.11	50.1	0.985	10.53	2.84

The negative ΔH° values indicate that the reactions are exothermic, and among all the catalysts, the composite catalyst showed the highest endothermic reaction for MB (ΔH° = −16.908 kJ/mol). All ΔS° values are also negative for all catalysts, indicative of decreasing entropy in the reactions due to more ordered dyes-catalysts interactions. Overall, these results highlight that the ZIF-8/ZnO/AC/NC composite enhances the thermodynamic favorability of dye degradation, particularly for MB, and suggests its potential for efficient, sustainable dye removal applications.

### Reusability and mechanism

**Fig. 10:**
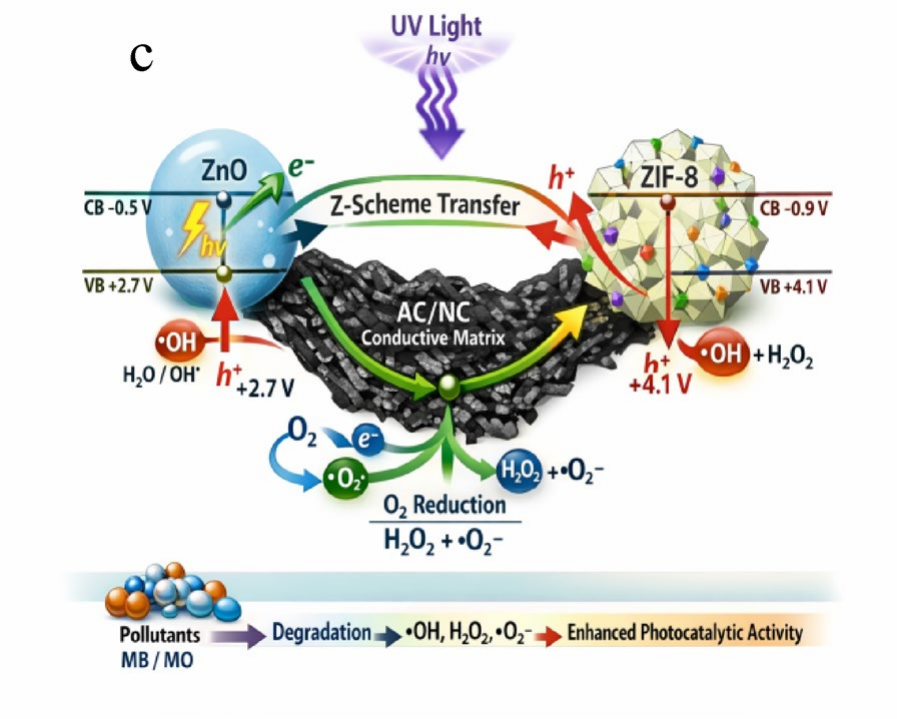
(**a**) and (**b**) Reusability of ZIF-8, ZnO, NC, AC and ZIF-8/ZnO/AC/NC composites, and (**c**) mechanism for the photocatalytic degradation of MB and MO using ZIF-8/ZnO/AC/NC.

The reusability results for both MB and MO (Fig. 10 and b) reveal a drop in efficiency over 5 consecutive cycles, but they exhibit good structural stability. In the case of MB, ZIF-8 slightly drops from 32% to 27.3%, whereas ZnO decreases from 53% to 48.9% indicating negligible extent of activity loss. AC and NC also experience modest decreases, respectively falling from 15% to 11.7% (18% to 14.7%).

**Table 6:** Thermodynamic parameters for the photocatalytic degradation of MB and MO using ZIF-8, ZnO, NC, AC and ZIF-8/ZnO/AC/NC composites.

Dye	Catalyst	ΔG° (kJ/mol)	ΔH° (kJ/mol)	ΔS° (J/mol·K)
MB	ZIF-8	−1.900	−6.768	−29.111
	ZnO	−0.303	−5.588	−17.878
	AC	−4.372	−12.680	−57.025
	NC	−3.822	−6.860	−35.711
	ZIF-8/ZnO/AC/NC	−6.088	−16.908	−36.924
MO	ZIF-8	−2.3805	−3.1715	−19.0050
	ZnO	−0.1008	−1.1395	−4.7667
	AC	−5.0220	−6.7333	−39.8070
	NC	−4.3721	−2.4418	−23.6187
	ZIF-8/ZnO/AC/NC	−4.8818	−8.3365	−12.4870

The composite (ZIF-8/ZnO/AC/NC) generally exhibits the superior stability, where approximately 87% removal efficiency remains in the fifth cycle of it compared with the value during the first cycle of 91.8%, reflecting very good durability to deactivation. The trend is same for MO: ZIF- 8, ZnO, AC and NC decreases from 28 to 22.7%, 49 to 45.3%,12 to 8.2% and15 to11.7% with respect of five cycles respectively. Also, the ZIF-8/ZnO/AC/NC composite possesses the best reusability from 87.4% to 83% and this indicates that the synergistic cooperation of ZIF-8, ZnO, AC and NC enhances better structural robustness as well as long-term activity compared to individual ones.

The ZIF-8/ZnO/AC/NC composite follows a Z-scheme-like charge transfer pathway mediated by the conductive AC/NC framework (Fig. 10c), which preserves strong redox potentials and enhances photocatalytic efficiency. Under UV illumination, ZnO (E_g_ = 3.2 eV, E_CB_ = −0.5 V, E_VB_ = + 2.7 V) acts as the primary photoactive phase, generating electron–hole pairs^62,63^. Unlike a conventional Type-II heterojunction, where electrons and holes migrate to lower energy levels and weaken redox ability, the AC/NC conductive matrix facilitates selective electron extraction from the ZnO conduction band and serves as an electron mediator and sink. Following the principles of mass action, the rapid removal and transfer of electrons from the ZnO CB shifts the equilibrium toward continuous charge separation, thereby increasing the lifetime and availability of highly oxidative holes in the ZnO (E_VB_ = + 2.7 V).

Simultaneously, ZIF-8 (E_CB_ ≈ −0.9 V, E_VB_ ≈ + 4.1 V) possesses a more positive valence band potential, which provides a strong thermodynamic driving force for the generation of hydroxyl radicals (bullet •OH) from surface-adsorbed water or hydroxide ions^64^. The high adsorption capacity of ZIF-8 ensures efficient mass transfer of pollutants (MB/MO) to the active sites, enabling direct oxidation by these high-potential holes^17^. Meanwhile, electrons transferred through the AC/NC conductive network are utilized for the multi-electron reduction of adsorbed oxygen to hydrogen peroxide H_2_O_2_ (+ 0.695 V) and other reactive oxygen species. This continuous electron extraction and utilization process, governed by the mass action principle, prevents charge accumulation and suppresses electron–hole recombination, thereby maintaining a sustained charge flow and significantly enhancing the photocatalytic degradation efficiency compared to pure ZnO.

Stepwise radical generation:

1 $$ZnO{\text{ }} + {\text{ }}h\nu {\text{ }} \to {\text{ }}ZnO{\text{ }}\left( {e^{ - } {\text{ }} + {\text{ }}h^{ + } } \right)$$

Photogenerated holes (h⁺) in the ZnO valence band oxidize surface-adsorbed water or hydroxide ions to produce hydroxyl radicals:

2 $$h^{ + } {\text{ }} + {\text{ }}H{}O/OH^{ - } {\text{ }} \to {\text{ }} \cdot OH$$

Electrons (e⁻) transferred from the ZnO conduction band through the AC/NC conductive network reduce adsorbed oxygen to superoxide radicals:

3 $$e^{ - } {\text{ }} + {\text{ }}O2{}{\text{ }} \to {\text{ }}O2{} \cdot ^{ - }$$

O₂•⁻ can further react with H⁺ to generate hydrogen peroxide (H₂O₂), which may decompose into additional •OH radicals:

4 $$O_{2} \cdot ^{ - } {\text{ }} + {\text{ }}2H^{ + } {\text{ }} + {\text{ }}e^{ - } {\text{ }} \to {\text{ }}H_{2} O_{2} {\text{ }} \to {\text{ }}2 \cdot OH$$

### Influence of water matrix components and interfering species

To investigate the photocatalytic degradation under realistic wastewater treatment conditions, the effects of common inorganic ions, dissolved organic matter and diverse water matrices were examined^65^. Multivalent cations, dissolved salts and natural organic matters are frequently encountered in industrial effluents and may have a remarkable effect on photocatalytic efficiency due to their competitive catalytic adsorption and scavenging of radicals. Consistent with prior literature, inorganic ions (Ca^2+^, Mg^2+^) had little inhibition but transition metal ions (Fe^3+^) greatly decreased degradative efficacy because of surface site blockage and recombination promotion (Table 7). Likewise, humic acid decreased degradation due to light absorption and hydroxyl radical scavenging (Table 8). In addition, photocatalytic activity was tested in different water matrices: distilled; tap; river water; and synthetic industrial waste-water (Table 9)^66^. High degradation efficiencies of above 80% were achieved by the catalyst even in complex media, indicating high resistance to matrix interference and application feasibility for degrading real wastewater. These results are in accordance with new contributions, highlighting the relevant effect of water matrix on the photo-catalytic efficiency in real conditions.

**Table 7:** Effect of interfering inorganic cations (5 mM) on the photocatalytic removal of MB and MO using ZIF-8/ZnO/AC/NC.

Ion (5 mM)	MB removal (%)	MO removal (%)
None	99.1	93.2
Na⁺	97.5	91.6
Ca²⁺	94.3	87.5
Mg²⁺	93.1	86.4
Fe³⁺	82.4	74.3

**Table 8:** Effect of Humic acid concentration on the photocatalytic removal of MB and MO using ZIF-8/ZnO/AC/NC.

Humic acid (mg/L)	MB removal (%)	MO removal (%)
0	99.1	93.2
5	95.4	88.6
10	91.2	83.5
20	85.3	76.8

**Table 9:** Effect of water matrix composition on the photocatalytic removal of MB and MO using ZIF-8/ZnO/AC/NC.

Water matrix	MB removal (%)	MO removal (%)
Distilled water	99.1	93.2
Tap water	94.8	89.3
River water	90.6	85.7
Simulated industrial wastewater	84.9	80.5

### Comparative photocatalytic removal efficiency of methylene blue (MB) and methyl orange (MO) using various catalysts reported in the literature and the present work

The individual materials (ZIF-8, ZnO, AC, and NC) showed moderate removal efficiencies for both MB and MO, with ZnO being the most active among them (Table 10). In contrast, the ZIF-8/ZnO/AC/NC composite achieved significantly higher removal (91.8% for MB and 87.4% for MO within 60 min at 0.3 g/L), demonstrating a strong synergistic effect between adsorption and photocatalysis. Although some reported catalysts reached slightly higher efficiencies, they often required longer reaction times or different dosages. Overall, the developed composite shows competitive and efficient dye removal performance under practical conditions.

**Table 10:** Comparative removal efficiency of methylene blue (MB) and methyl orange (MO) using individual components and the ZIF-8/ZnO/AC/NC composite, along with previously reported photocatalysts, including catalyst dosage and reaction time.

Dye	Catalyst	Removal %	Dose of catalyst (g)/L	Time	Ref
MB	ZIF-8	32	0.3	60	This work
	ZnO	53	0.3	60	This work
	AC	15	0.3	60	This work
	NC	18	0.3	60	This work
	ZIF-8/ZnO/AC/NC	91.8	0.3	60	This work
	TiO_2_NPs@ZIF-8	93	0.01	120	^67^
	ZIF-8@Ti_3_ C_2_ MXene	95	0.02	120	^68^
	rGO/ZnO-flowers	97.1	0.02	105	^69^
	ZnO/AC	100	0.3	180	^70^
	TiO2/activated carbon	99.43	0.4	60	^71^
	Fe-doped ZnO/Nanocellulose	94.21	0.15	90	^72^
MO	ZIF-8	28	0.3	60	This work
	ZnO	49	0.3	60	This work
	AC	12	0.3	60	This work
	NC	15	0.3	60	This work
	ZIF-8/ZnO/AC/NC	87.4	0.3	60	This work
	Ppy/Ag-ZnO	91.11	0.07	90	^73^
	ZnO/TiO _2_	98.6	0.02	300	^74^
	TiO _2_ /cellulose fabric	99.5	0.03	100	^75^
	TiO _2_ @ZIF-8	99	0.2	100	^76^
	Cu supported on ZnO	99	0.15	90	^77^

## Conclusion

The ZIF-8/ZnO/AC/NC composite demonstrated outstanding photocatalytic capability for degrading MB and MO dyes, significantly outperforming each individual component. This superior performance originates from the rational integration of a photoactive semiconductor (ZnO), a highly porous MOF (ZIF-8), conductive activated carbon, and sustainable nanocellulose into a single multifunctional heterostructure, representing a novel and environmentally compatible quaternary photocatalytic system. The composite achieved optimal degradation efficiencies of 91.8% for MB and 87.4% for MO under mild operating conditions (pH 7, 0.3 g/L catalyst dosage, 30 °C, and 60 min UV irradiation), demonstrating its high efficiency and operational feasibility. Langmuir isotherm analysis revealed high monolayer adsorption capacities (qmax = 53.0 mg/g for MB and 50.1 mg/g for MO), confirming strong and favorable adsorption on the composite surface. The hierarchical porous architecture provided by activated carbon and nanocellulose significantly enhanced dye diffusion, adsorption, and accessibility to active sites, while the synergistic interaction between ZnO and ZIF-8 promoted efficient charge separation and reactive oxygen species generation. Kinetic modeling confirmed a pseudo-second-order mechanism, and thermodynamic parameters indicated that the reactions proceeded spontaneously and exothermically, confirming the energetic favorability of the process. Reusability tests further demonstrated the composite’s robustness, maintaining high performance across five consecutive cycles with minimal efficiency loss, highlighting its excellent structural stability and long-term operational durability. These findings confirm that the composite offers a reliable and sustainable solution for repeated wastewater treatment applications. From a practical perspective, the use of low-cost biomass-derived nanocellulose and activated carbon, combined with scalable synthesis methods, enhances the feasibility of large-scale implementation in real wastewater treatment systems. Furthermore, the multifunctional design strategy presented in this study provides an effective framework for developing next-generation hybrid photocatalysts with improved efficiency, sustainability, and stability. Future research should focus on evaluating the composite under visible light irradiation and solar-driven conditions, investigating its performance in real industrial wastewater, and optimizing its structural and electronic properties to further enhance photocatalytic efficiency and scalability. Additionally, exploring magnetic modification or immobilization strategies may facilitate catalyst recovery and continuous flow applications, advancing its practical deployment in industrial wastewater remediation.

## Data Availability

Data will be available upon request.
